# Beyond Proteostasis: Lipid Metabolism as a New Player in ER Homeostasis

**DOI:** 10.3390/metabo11010052

**Published:** 2021-01-14

**Authors:** Jiaming Xu, Stefan Taubert

**Affiliations:** 1 Graduate Program in Cell and Developmental Biology, The University of British Columbia, Vancouver, BC V6T 1Z4, Canada; cxu@cmmt.ubc.ca; 2 Centre for Molecular Medicine and Therapeutics, The University of British Columbia, Vancouver, BC V5Z 4H4, Canada; 3 Healthy Starts Theme, British Columbia Children’s Hospital Research Institute, Vancouver, BC V5Z 4H4, Canada; 4 Department of Medical Genetics, The University of British Columbia, Vancouver, BC V5Z 4H4, Canada

**Keywords:** lipid bilayer stress, unfolded protein response, unsaturated fatty acid, endoplasmic reticulum, phosphatidylcholine, lipidomics

## Abstract

Biological membranes are not only essential barriers that separate cellular and subcellular structures, but also perform other critical functions such as the initiation and propagation of intra- and intercellular signals. Each membrane-delineated organelle has a tightly regulated and custom-made membrane lipid composition that is critical for its normal function. The endoplasmic reticulum (ER) consists of a dynamic membrane network that is required for the synthesis and modification of proteins and lipids. The accumulation of unfolded proteins in the ER lumen activates an adaptive stress response known as the unfolded protein response (UPR-ER). Interestingly, recent findings show that lipid perturbation is also a direct activator of the UPR-ER, independent of protein misfolding. Here, we review proteostasis-independent UPR-ER activation in the genetically tractable model organism *Caenorhabditis elegans*. We review the current knowledge on the membrane lipid composition of the ER, its impact on organelle function and UPR-ER activation, and its potential role in human metabolic diseases. Further, we summarize the bi-directional interplay between lipid metabolism and the UPR-ER. We discuss recent progress identifying the different respective mechanisms by which disturbed proteostasis and lipid bilayer stress activate the UPR-ER. Finally, we consider how genetic and metabolic disturbances may disrupt ER homeostasis and activate the UPR and discuss how using -omics-type analyses will lead to more comprehensive insights into these processes.

## 1. Introduction

Within the eukaryotic cell, the endoplasmic reticulum (ER) is a dynamic membrane network involved in many essential cellular processes. The rough ER has membrane-bound ribosomes and is a site for the synthesis, maturation, and modification of more than one-third of the human proteome. The smooth ER functions in lipid and steroid hormone biosynthesis and xenobiotic detoxification. Although ER homeostasis is critical, it is prone to various cellular stressors such as intracellular Ca^2+^ imbalance, viral infection, changes in redox environment, and hypoxia, all of which trigger a state known as ER stress [1]. Moreover, in highly proliferative or secretory cells, the influx of large amounts of nascent proteins into the ER can temporarily overburden the folding machinery, leading to endogenous ER stress [2]. Prolonged ER stress can compromise cellular function and viability and lead to or exacerbate many human diseases, including cancer, diabetes, and neurodegenerative conditions [3]. 

**Figure 1:**
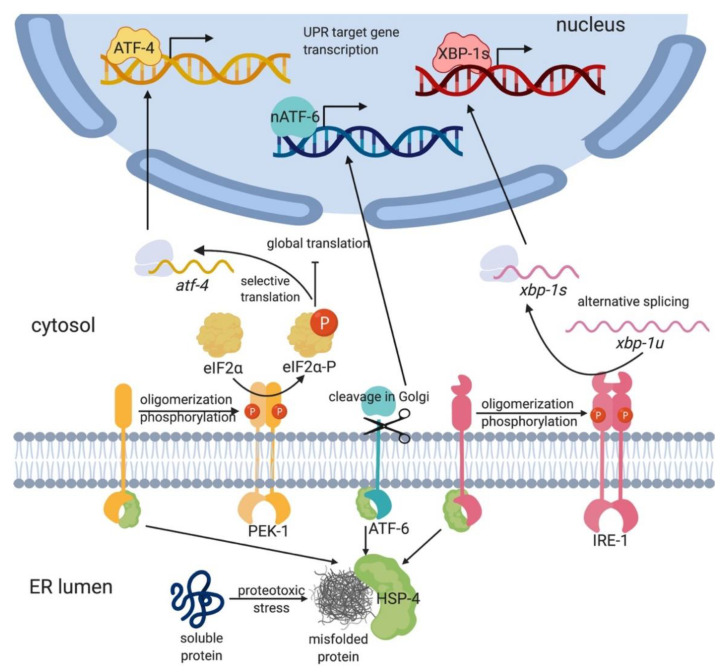
Overview of the canonical endoplasmic reticulum unfolded protein response (UPR-ER) pathways. In higher eukaryotes, upon sensing misfolded proteins by HSP-4/BiP, the three UPR-ER branches—IRE-1α, PEK-1, and ATF-6—are activated to mount distinct and collective downstream transcriptional and translational programs to promote protein folding, processing, and secretion, thereby reducing the load of misfolded proteins in the ER lumen and alleviating ER stress. Abbreviations: ATF-4: Activating Transcription Factor 4; ATF-6: Activating Transcription Factor 6; eIF2α: Eukaryotic Initiation Factor 2α; HSP-4/BiP: heat shock protein 4/ Binding immunoglobulin protein; IRE-1α: Inositol-Requiring-Enzyme 1α; PEK-1: human PERK kinase homolog; UPR: unfolded protein response; *xbp-1*: X-box Binding Protein homolog 1. (Figure created with Biorender.com, Toronto, ON, Canada).

To ensure viability and proper cellular function, cells have evolved a conserved adaptive mechanism to restore ER homeostasis under stress: the ER unfolded protein response (UPR-ER; Figure 1) [4,5]. In higher eukaryotes, the UPR-ER is composed of three parallel ER stress sensing and transducing branches: the Inositol-Requiring-Enzyme 1α (IRE-1α, also known as Endoplasmic Reticulum to Nucleus signaling 1 or ERN1 in mammals) branch [2]; the protein kinase RNA-like ER kinase (PERK; also known as human PERK kinase homolog, PEK-1; or Eukaryotic Translation Initiation Factor 2 Alpha Kinase 3 or EIF2AK3) branch [6]; and the Activating Transcription Factor 6 (ATF-6) branch [7] (Figure 1). These three sensors are embedded in the ER membrane with a single-pass transmembrane domain, which connects a luminal sensor domain to a cytosolic effector domain. This modular design enables these sensors to communicate the input stress signal to transcriptional and translational machineries for effector output. Together, they attenuate ER stress by reprograming transcription and translation to promote protein folding, degradation, and transport, as well as lipid synthesis and remodeling [8]. Alternatively, if ER stress is not resolved, the UPR-ER switches from promoting survival and adaptation to triggering apoptosis [8]. 

The nematode roundworm *Caenorhabditis elegans* (*C. elegans*) has emerged as a useful model to study a large variety of cellular processes. Genome sequencing and comparative proteomics studies have revealed that more than 80% of the *C. elegans* proteome has human homologs [9]. Moreover, many genetic pathways that were initially discovered in worms also exist in other species [10], including those involved in lipid metabolism and the UPR-ER. For example, the three core UPR-ER signal transducers display high levels of conservation between *C. elegans* and mammals, including humans. Furthermore, 237 of the 471 curated *C. elegans* lipid metabolism genes are conserved in humans and/or other model organisms, and 71 of these are implicated in human metabolic diseases [11,12]. *Vice versa*, as with all model systems, there are limitations to the use of *C. elegans*. Pertinent to the study of the UPR-ER, these include (i) a relative lack of accessibility to manipulation with drugs, which sometimes fail or work only at extremely high doses (e.g., chemical chaperones, see below); (ii) differences in membrane lipid composition, as *C. elegans* features very low to no cholesterol in its cellular and subcellular membranes; and (iii) mechanistic differences concerning nuanced roles of the UPR-ER, such as regulated IRE1-dependent decay of mRNA (RIDD; see below). Nevertheless, because of the high level of conservation of the core UPR-ER pathways and of (lipid) metabolism, and because of the powerful genetic and genomic tools including forward and reverse genome-wide screens, *C. elegans* is an excellent model that has provided important new insights into the mechanistic basis of UPR-ER regulation under different stress conditions, including lipotoxicity [13,14,15,16,17,18,19,20,21,22].

In this review, we summarize evidence that supports the bi-directional interplay between lipid metabolism and UPR-ER activation in different species with an emphasis on *C. elegans*, focusing on the differences from proteotoxicity-induced UPR-ER. Additionally, we explore the potential of -omics approaches to delineate how metabolic disturbances might activate the UPR-ER in *C. elegans*, and how this could deepen our understanding of lipid-disturbance-induced UPR-ER in the pathophysiology of human metabolic diseases.

## 2. The Animal UPR-ER Is Composed of Three Branches

### 2.1. IRE1 Is the Most Highly Conserved and Ancient UPR-ER Transducer

IRE1 (IRE-1 in *C. elegans*) is an ER resident protein evolutionarily conserved from yeast to humans. It was first described in the yeast *Saccharomyces cerevisiae* as essential for growth in the absence of inositol [23] and emerged as the sole ER stress transducer in yeast [24,25]. Allosteric IRE1 activation involves the protein chaperone Binding immunoglobulin protein (BiP; also known as 78-kDa glucose-regulated protein (GRP78) or heat shock protein 5α (HSP5α), and as heat shock protein 4 (HSP-4) in *C. elegans*). Misfolded proteins bind to BiP/HSP-4, which causes its dissociation from IRE1’s luminal domain (LD; Figure 1) [26]. The dissociation of BiP/HSP-4 from the LD triggers the formation of IRE1 dimers and higher-order oligomers, leading to robust trans-autophosphorylation [26]. Phosphorylated and active IRE1 then uses its endoribonuclease activity to excise a 26-base-pair intron in a target mRNA encoding the transcription factor ATF/3’,5’-cyclic adenosine monophosphate (cAMP) responsive element binding protein 1 (CREB1) homolog (HAC1; in yeast) or X-box binding protein 1 (XBP1 or XBP-1; in metazoans) [27]. The excision and re-ligation shift the open reading frame, resulting in the translation of the spliced *XBP1* mRNA (*sXBP1*; *xbp-1s* in *C. elegans*), which is more active and stable than the unspliced *XBP1* mRNA (*uXBP1*; *xbp-1u* in *C. elegans*) [28,29]. After translation, HAC1/sXBP1/XBP-1s translocates to the nucleus and initiates the transcription of cytoprotective genes involved in protein folding, translocation, and glycosylation; redox metabolism; autophagy; cell wall synthesis; vesicular trafficking; ER-associated degradation (ERAD); and lipid/inositol metabolism [8,24,30,31]. 

IRE1 has alternative roles besides *XBP1* splicing. IRE1 mediates the degradation of a subset of mRNAs and microRNAs via a process termed regulated IRE1-dependent decay (RIDD) in *Drosophila melanogaster* [32], mammalian cells [33], and *Schizosaccharomyces pombe* [34]. The majority of mRNAs targeted by RIDD encode ER-resident proteins, whose presence would present additional challenges to an already-stressed organelle [32,33,34]. Mechanistically, IRE1 adopts higher-order oligomeric structures to splice XBP1/HAC1, whereas monomeric IRE1 is sufficient to activate RIDD [35]. Intriguingly, evidence for RIDD in *C. elegans* is currently lacking. Moreover, independently of the endoribonuclease domain, IRE1 can initiate ERAD, which targets terminally misfolded proteins for degradation in the cytoplasm by the ubiquitin–proteasome system (UPS) [36]; this role is conserved from yeast to mammals, including in *C. elegans* [18].

*IRE1* null mutant yeasts are viable under normal growth conditions but not in the presence of ER-stress-inducing drugs such as tunicamycin and β-mercaptoethanol [24]. Similarly, *C. elegans ire-1* or *xbp-1* loss-of-function mutants fail to survive ER stress conditions and pathogen infection [37,38]. Although *C. elegans ire-1* mutant worms do not exhibit gross developmental defects, recent studies found that *ire-1*, independently of *xbp-1*, is required for neuronal development [39]. In contrast, *D. melanogaster Xbp1* null [40] and mouse *Ire1α−/−* and *Xbp1−/−* null mutations are lethal [41]. This variation in phenotypic manifestation highlights the importance of IRE1 as a UPR-ER sensor and also indicates a spatiotemporally unequal demand for IRE1 functions during organism development.

### 2.2. The PERK/PEK-1 Branch of the UPR-ER Reprograms Translation

In higher eukaryotes, including *C. elegans*, the UPR-ER is more complex as it includes additional ER stress transducers besides IRE1 (Figure 1). PERK/PEK-1, like IRE1, is a transmembrane kinase whose luminal domain dissociates from BiP upon sensing misfolded proteins, whereupon PERK forms dimers and undergoes auto-phosphorylation [42]. Active PERK then phosphorylates the eukaryotic translation initiation factor-2 (eIF2α) and thus transiently inhibits general protein translation initiation, thereby reducing ER proteostasis stress. However, phosphorylation of eIF2α also enables the selective translation of activating transcription factor 4 (ATF4), which upregulates a subset of UPR genes, including the apoptosis-inducing CCAAT/Enhancer Binding Protein (C/EBP) homologous protein (*CHOP*; also known as also known as growth arrest- and DNA damage-inducible gene 153, *GADD153*) and the growth arrest and DNA damage-inducible gene 34 (*GADD34*) [35], restoring balance by dephosphorylating eIF2α [43]. Whereas the downstream actions of ATF4 are not well known in *C. elegans*, worm ATF-4 resembles human ATF4 in gene structure and regulation by upstream open reading frames [44] and in its response to general translational inhibition [45], implying strong functional similarity.

PERK plays important roles in animal development. Although *C. elegans pek-1* single mutant worms show no noticeable phenotypes, a *pek-1;ire-1* double mutant arrests at the second larval (L2) stage due to intestinal degeneration [46]. Consistent with its function as an important UPR-ER sensor, absence of *pek-1* renders worms hypersensitive to ER-stress-inducing toxins such as tunicamycin, and loss of *PERK* in cultured mammalian cells causes similar phenotypes [46,47]. Similarly, *Perk-/-* mice, although viable, develop progressive diabetes mellitus because they amass misfolded proteins in the highly secretory pancreatic β-cells [48]. In addition, homozygous loss of *Perk* in humans causes onset of type 1 diabetes during infancy [49]. Collectively, these observations in different organisms highlight the importance of PERK in alleviating exogenous and endogenous ER stress.

### 2.3. ATF6 Is a Parallel Sensor that Modulates UPR-ER Pathways

Animals feature a third UPR-ER pathway consisting of ATF6 (ATF6α and ATF6β in mammals). Like IRE1 and PERK, ATF6α is an ER transmembrane protein (Figure 1). However, ATF6α is different in its domain architecture and mode of action [2]. In the absence of stress, the luminal domain of ATF6α associates with BiP, shielding a Golgi localization sequence within ATF6α and thus anchoring it to the ER membrane. Upon ER stress, BiP dissociates from ATF6α, which translocates to the Golgi, where it is proteolytically processed by site 1 and site 2 proteases (S1P and S2P). This releases the cytosolic, N-terminal basic leucine zipper (bZIP) transcription factor domain (ATF6-p50), which then translocates to the nucleus and upregulates UPR-ER genes [2]. Specifically, ATF6α is required to express the *XBP1* mRNA, which is then spliced by activated IRE1 [29,50], leading to synergistic UPR-ER activation by two distinct branches. Moreover, ATF6α can function independently or heterodimerize with sXBP1 to promote the expression of ERAD components, chaperones, and UPR mediators, including BiP and XBP1 in mammals [51,52] and *C. elegans* [53]. Mammalian ATF6α also modulates *XBP1* splicing and promotes the expression of the ATF4 target *CHOP* in response to chronic ER stress [54], suggesting that ATF6α may function as a modulator of the IRE1 and PERK branches. In *C. elegans*, the ATF6α homolog ATF-6 regulates few inducible UPR-ER genes but is required to express many constitutively expressed UPR-ER genes; this distinguishes it from IRE-1 and PEK-1, which primarily upregulate inducible UPR-ER genes following ER stress, thus highlighting a distinct function for ATF-6 [46]. Consistently, *atf-6* mutant worms do not display overt developmental phenotypes or sensitivity to tunicamycin [18], whereas *ire-1;atf-6* and *xbp-1;atf-6* double mutant worms show synthetic lethality [18,46,54].

Some mammalian species encode two ATF6 isoforms. Atf6α regulates stress recovery in vitro [54] and in vivo [51], but its target genes vary in different cell types [55]. In unstressed conditions, the effect of losing either Atf6α or Atf6β is mild [51,56], whereas losing both results in embryonic lethality in mice [51]. This is in line with the observation that *C. elegans* ATF-6 regulates constitutive UPR-ER genes and enables coping with endogenous ER stress during development [18]. Thus, the mammalian ATF6 branch likely possesses both conserved and distinct roles.

## 3. Membrane Lipids Are Critical for Normal ER Function

In mammals, lipids are categorized into six major classes: fatty acyls, glycerolipids, glycerophospholipids, sterol lipids, prenol lipids, and sphingolipids [57,58]. *C. elegans* has a similar lipid composition but with some additional lipid subclasses [11]. In all species, lipids are essential as they serve as a source of energy, mediate signal transduction, and form cellular and organellular membranes. In addition to the well-known functions of membranes, such as providing a physical barrier, membrane lipids and their modifications actively regulate cellular and subcellular trafficking [59]. Membrane lipids belong to three main categories: phospholipids, sphingolipids, and sterols (cholesterol in mammals, ergosterol in yeast) [60]. They vary in structure and distribution, and this diversity is maintained from the organismal level to the subcellular and membrane subdomain levels. For example, at the organelle level, lipidomic analysis in mammalian cells revealed that each organelle has a distinct membrane lipid profile [61]. Phosphatidylcholine (PC) is most abundant in the ER membrane (57%) and less abundant in the inner mitochondrial membrane (41%), whereas cardiolipin (CL) is present only in mitochondrial membranes [61,62]. Maintaining this unique quantitative and qualitative composition is critical for the normal functions of each organelle and, therefore, the cellular function and overall health of an organism. For example, reducing CL levels by blocking phosphatidic acid transfer causes cytochrome c release and apoptosis [63]. Moreover, in vivo studies show that blocking phosphatidylethanolamine (PE) synthesis at the mitochondrial inner membrane causes embryonic lethality in mice [64]. Links between different diseases and different lipid classes have been reviewed in detail elsewhere [65].

## 4. Bidirectional Interplay between Lipid Metabolism and the UPR-ER

**Figure 2:**
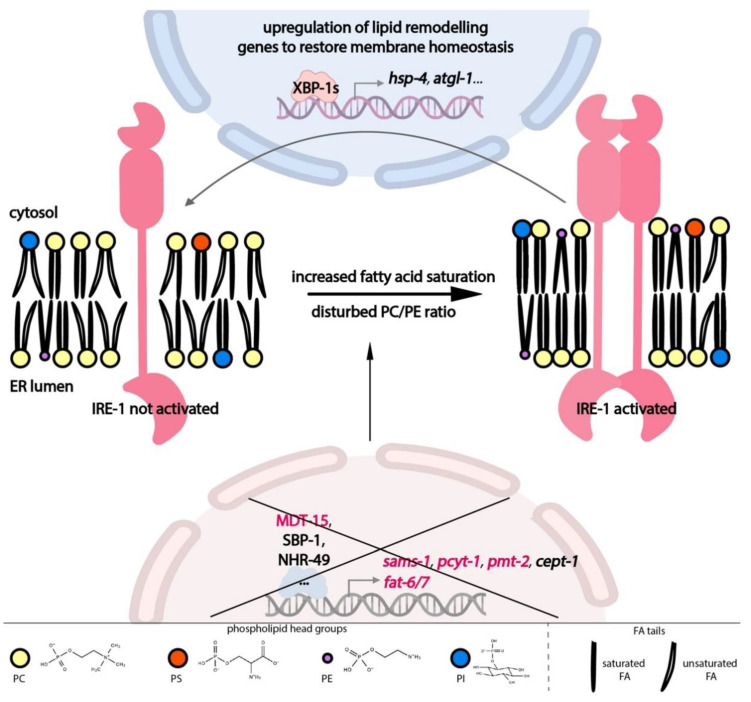
Overview of the bidirectional interplay between lipid metabolism and the IRE-1 branch of the UPR-ER in *Caenorhabditis* *elegans*. Disturbed ER membrane lipid composition is caused by the loss of *mdt-15* or *fat-6/7*, which cause increased FA saturation, or by the loss of *mdt-15*, *sams-1*, *pcyt-1*, or *pmt-2*, which cause disturbed PC/PE ratios. All these disturbances are direct triggers for IRE-1 activation, i.e., independent of protein misfolding. Activated IRE-1 upregulates compensatory genes, which remodel lipid metabolism and restore a proper lipid environment in the ER. Genes colored in red have been experimentally shown to cause IRE-1 activation in *C. elegans* when inactivated. Abbreviations: *atgl-1*: adipose triglyceride lipase; *cept-1*: choline/ethanolaminephosphotransferase; FA: fatty acid; *fat-6/-7*: fatty acid desaturase 6/7; *hsp-4*: heat shock protein 4; IRE-1: IRE1 kinase related; MDT-15: mediator 15; NHR-49: nuclear hormone receptor 49; PC: phosphatidylcholine; *pcyt-1*: phosphocholine cytidylyltransferase; PE: phosphatidylethanolamine; PI: phosphatidylinositol; *pmt-2*: phosphoethanolamine methyltransferase; PS: phosphatidylserine; *sams-1*: S-adenosyl methionine synthetase; SBP-1: sterol regulatory element binding protein; XBP-1: X-box binding protein homolog. (Some parts of the image were created with BioRender.com, Toronto, ON, Canada).

It is now clear that the UPR-ER’s importance goes beyond proteostasis. In line with the ER’s dual function in protein and lipid production, membrane lipid imbalance can directly activate the UPR-ER. In turn, the UPR-ER directly upregulates compensatory pathways to restore lipid homeostasis. Thus, the UPR-ER is intricately linked to lipid metabolism and homeostasis both upstream and downstream, as outlined below (Figure 2).

Feedback from different lipid metabolic pathways modulates ER homeostasis through the UPR-ER sensors (Figure 2), including inputs from fatty acid (FA) tails and from lipid head groups; this type of ER stress is also known as lipid bilayer stress (LBS). Diets enriched in long-chain saturated FAs induce the UPR-ER [66,67], as does the inactivation of the FA desaturation machinery, which produces unsaturated FAs in yeast, worms, and human cells [68,69,70]. In *C. elegans*, RNA interference (RNAi) knockdown of the stearoyl-CoA desaturases (SCDs) fat-6 and fat-7 activates the transcription of an IRE-1-branch-specific hsp-4/BiP reporter. Dietary supplementation with oleate, a mono-unsaturated FA (MUFA), is sufficient to suppress the activation of hsp-4 from SCD knockdown [71], indicating that adequate membrane lipid unsaturation is required to prevent ER stress and concomitant UPR-ER activation in *C. elegans*.

Similarly, the nature of a lipid’s head group is also important for ER homeostasis (Figure 2). RNAi knockdown of Mediator subunit 15 (mdt-15), a conserved transcriptional co-regulator, leads to a significant reduction in PC levels and activates the IRE-1 and PEK-1 branches [71]. This activation is partially suppressed by choline supplementation [13], indicating that appropriate PC levels are required for ER homeostasis. Indeed, abnormal PC/PE ratios caused by deleting or inactivating any of the PC synthesis genes encoding S-adenosyl methionine synthetase (sams-1), phosphocholine cytidylyltransferase (pcyt-1), and phosphoethanolamine methyltransferase (pmt-2) also cause UPR-ER activation in *C. elegans* [13,17,71], in yeast [72], and in mice [73]. These studies suggest that UPR-ER sensors can sense different types of LBS, i.e., inputs, across species.

In terms of output, UPR-ER sensors are critical for maintaining lipid homeostasis under lipotoxic stress. Recent work has provided molecular evidence for the regulatory role of UPR-ER branches in lipid metabolism in *C. elegans* (Figure 2). In *C. elegans*, *ire-1* and its downstream target *hsp-4* are required for fasting-induced fat droplet hydrolysis through the actions of fasting-dependent lipases [74]. Additionally, in a *pmt-2* mutant with defective PC synthesis, *ire-1* is required to induce lipid metabolism genes such as lipid droplet-associated lipase adipose triglyceride lipase (*atgl-1*) [75], which is necessary for TG hydrolysis. In contrast, this activation is absent in tunicamycin-induced proteotoxic stress [76]. Interestingly, similar to the lethality rescue by ectopic *Xbp1* in flies [77], intestinal remodeling of the lipidome can be achieved by neuron-specific *xbp-1s* overexpression in *C. elegans*. This occurs via tyramine as an inter-tissue signalling molecule, which thus contributes to overall organismal proteostasis and increased life span [19,21]. Lastly, transcriptome studies show that *C. elegans* IRE-1, PEK-1, and ATF-6 differentially upregulate specific sets of genes in a *pmt-2* deletion mutant, with about half of the 1069 lipid-stress-specific genes being controlled by two or more branches, suggesting combinatorial roles of UPR-ER sensors during PC depletion [17]. Overall, these results show that the UPR-ER is an adaptive stress response that is a central lipid metabolism regulator in worms.

## 5. The Role of Lipid Metabolism and ER Homeostasis in Human Diseases

With over 130 lipid metabolism genes implicated in human genetic diseases, lipid homeostasis plays a pivotal role in human health [78,79]. For example, human SCD is a highly conserved, ubiquitously expressed, ER-localized ∆9-desaturase that converts saturated FAs into MUFAs. The human *SCD* gene consists of six exons and five introns and is found on chromosome 10. Human *SCD* is implicated in various pathological processes, such as eating disorders, cardiovascular disease, and obesity [80,81,82,83]. It is expressed in numerous tissues and regulated by manifold inputs, factors, and pathways. For example, *SCD*’s transcriptional control is complex, including regulation by PUFAs, cholesterol, vitamin A, hormones, developmental signals, temperature changes, and the presence of metals and phenolic compounds [83,84,85,86,87].

The connections between lipid metabolism and the UPR-ER have attracted increasing attention, particularly in oncological settings. Different aspects of FA metabolism, including de novo FA synthesis, FA uptake, FA degradation, and phospholipid metabolism are altered in many cancer types [88,89,90]. For instance, human *SCD* is critical for growth in many different cancers, particularly in lung cancer, where its expression is inversely correlated with patient survival outcome [91]. In hypoxia, cancer cells deprived of oxygen are unable to maintain proper lipid unsaturation via endogenous SCD activity. Instead, they rely on increased uptake of exogenous unsaturated lipid species, mainly MUFAs, through the upregulation of the FA importer *CD36*; indeed, the lack of unsaturated FAs activates *IRE1α*-dependent cell death [92,93]. Yet, despite the tumor-suppressing role of terminal UPR-ER, other studies suggest that all three adaptive UPR-ER branches support tumor growth in vivo [94], and this has fueled the development of anticancer drugs targeting these branches [95,96].

Besides oncological settings, disturbances to local ER membrane lipid composition also correlate with atherosclerotic lesions [97] and obesity in humans [98,99]. Moreover, the altered composition of ER lipids has profound secondary influences. For example, an accumulation of PS in the ER results in disturbed phosphatidylinositol 4-phosphate (PI4P) metabolism and distribution and is a known cause for Lenz–Majewski syndrome [100]. Therefore, disturbances to ER lipid composition are not only a local phenomenon, but lead to malfunctions in other organelles and overall cellular defects, because the ER regulates the lipid composition of other organelles through direct contact sites [101,102] and vesicular trafficking [102].

## 6. Lipotoxicity Activates the UPR-ER through a Distinct Mechanism from Proteotoxicity

The mechanistic details of how membrane lipid perturbation is sensed by the UPR-ER have begun to emerge over the last few years. Early studies showed that UPR-ER induction by saturated FAs in yeast [68] can be suppressed by chemical chaperones, such as 4-phenyl butyrate (4-PBA), which promote protein folding. Similarly, in studies with obese mouse models with steatotic livers, chemical chaperones such as 4-PBA and tauro-ursodeoxycholic acid (TUDCA) also resolve obesity-induced hepatic lipid accumulation [103]. These observations favor a model of membrane lipid disturbances as an indirect activator of the UPR-ER, upstream of proteotoxicity; in other words, through protein misfolding [35].

However, the role of chemical chaperones is more complex than facilitating protein folding. 4-PBA reduces Ire1p levels instead of unfolded protein load, providing an alternative interpretation of the above results [104]. Furthermore, 4-PBA and TUDCA have functions beyond protein refolding, such as reducing lipid accumulation [105] and membrane cholesterol levels [106], as well as restoring ER lipid fluidity and calcium permeability [107]. Therefore, interpreting results from experiments with chemical chaperones is challenging.

Indeed, later work from several groups offers an alternative view on the mechanisms underlying lipotoxicity-induced UPR-ER. For example, long-chain saturated FAs reduce Ca^2+^ in the ER of hepatic cells [108]. Consistently, lipid bilayer stress (LBS) caused by altered lipid saturation or phospholipid head group composition in the liver of obese mice inhibits the sarco-/endoplasmic reticulum calcium ATPase (SERCA), thus reducing Ca^2+^ in the ER [73]. However, the effect of Ca^2+^ level changes in the ER is not limited to protein folding capacity changes, as ER Ca^2+^ homeostasis is also implicated in lipid storage in cultured cells and flies [109]. Moreover, comparative proteomics of ER from the liver of obese mice that experienced LBS did not show significant alterations in the chaperone content compared to the lean mouse control, whereas an enrichment in lipid metabolism enzymes was observed [73]. This suggests that, in addition to the idea that Ca^2+^ changes activate the UPR-ER by reducing the load of misfolded proteins, lipotoxicity-induced changes in ER Ca^2+^ content may also activate the UPR-ER via concurrent lipid alterations. 

Furthermore, several lines of evidence indicate that, parallel to proteotoxicity-induced UPR-ER (also referred to as UPR-ER^PT^), disturbances to lipid composition directly induce the UPR-ER; this is termed lipid bilayer stress (LBS)-induced UPR-ER (UPR-ER^LBS^). First, different IRE1 cluster formation in cells experiencing proteotoxic and lipotoxic stress provides indirect evidence that LBS activates UPR-ER through a mechanism different from protein misfolding. Specifically, in HeLa cells transfected with an IRE1-GFP(green fluorescent protein) fusion reporter, tunicamycin-induced ER stress caused IRE1 to form distinctive puncta, whereas palmitate-induced ER stress caused diffuse IRE1 distribution throughout the ER membrane [110]. Similar findings were reported in yeast where IRE1 formed clustered puncta in response to dithiothreitol (DTT)-induced proteotoxic ER stress, whereas such puncta were absent during UPR-ER^LBS^ in opi3 mutants that failed to synthesize PC [13]. Furthermore, 4-PBA was able to attenuate tunicamycin-induced UPR-ER^PT^ but not opi3-deletion-induced UPR-ER^LBS^ [13]. Second, additional evidence confirmed that LBS directly activates the UPR-ER through a novel, membrane-based mechanism that is independent of protein misfolding. In *C. elegans*, increased lipid saturation or decreased PC content activates the UPR-ER via the IRE-1 branch (Figure 2). Critically, this is independent of protein misfolding, as shown by the lack of aggregates of a misfolding-prone protein reporter [71]. Third, the UPR-ER is activated in yeast by reduced PC and PI content [111] and in cell lines by increased lipid saturation [69] even when the luminal misfolded protein sensing domain of IRE1 or PERK is deleted. Thus, the UPR-ER^LBS^ is molecularly separable from protein-misfolding-induced UPR-ER. Fourth, there are important mechanistic differences in how proteotoxicity and lipotoxicity activate IRE1 in yeast. Overexpressing the luminal domain of IRE1 (IRE1^LD^) completely attenuated proteotoxicity (tunicamycin) induced UPR-ER, whereas lipotoxicity (in opi3 mutants) induced UPR-ER^LBS^ was only partially attenuated by overexpressing either IRE1-LD or IRE1ΔLD; this suggests that lipotoxicity requires a novel activation mechanism of IRE1 [13]. Moreover, this study pinpointed Arginine 537 at the interface of the amphipathic and transmembrane helices in IRE1 as required for UPR-ER^LBS^ but not UPR-ER^PT^. Additionally, in yeast, transcriptomic analysis identified a novel subset of genes only induced by lipotoxicity in opi3 mutants; this further differentiates UPR-ER^PT^ and UPR-ER^LBS^ [13]. Similarly, in *C. elegans*, activation of lipophagy by the UPR-ER is sufficient to drive lipid depletion and restructure ER morphology, thus promoting life-span extension. This occurs independently of chaperone induction [22], providing further evidence that proteostasis and lipid homeostasis are separate UPR-ER dependent processes. Collectively, these studies demonstrate that the UPR-ER can be activated directly by two parallel mechanisms: (i) by sensing aberrant protein folding and processing or (ii) via altered membrane lipid composition, with modest activation in both parallel pathways leading to higher synergistic IRE1 activation. Such a dual sensing and response mechanism is consistent with ER’s dual function in protein and lipid synthesis and processing.

## 7. Crosstalk between Proteotoxicity- and Lipotoxicity-Induced UPR-ER

Intriguingly, despite clear differences, the separation between proteotoxicity- and lipotoxicity-activated UPR is not absolute. In S. cerevisiae, lipid imbalance can be observed concomitantly with disturbed ER proteostasis [35,112,113]. For example, chronic palmitate exposure results in disrupted ER lipid rafts and causes protein overload in mouse β-cell lines [114], providing a mechanistic framework to explain how lipotoxicity leads to proteotoxicity. Indeed, membrane lipid composition affects the sorting of many proteins to different organelles in yeast and mammalian cells, as the properties of protein transmembrane domains interact differentially with the properties of the target membrane bilayer, e.g., the thickness and chemical properties [115]. On the other hand, supplementation with oleic acid reduced disease phenotypes associated with the expression of exogenous poly-Q40, an aggregating polyglutamine peptide, in *C. elegans* [20]. This suggests that changes in the lipidome are sufficient to improve protein homeostasis through mechanisms other than chaperone induction. Molecular evidence also supports the importance of lipid homeostasis in directly maintaining proteostasis. In vitro biophysical assays have established the role of different classes of lipids as catalysts or inhibitors for protein folding. Anionic phosphatidylserine (PS) accelerates human amylin protein aggregation, whereas cholesterol attenuates it [116]. In addition, membrane-vesicle-based studies show that PE functions as a lipid chaperone that enables the folding of the Escherichia coli membrane protein lactose permease [117]. Based on these studies, we conclude that rather than a downstream response, lipotoxicity can induce or exacerbate proteotoxicity directly, determined by the inherent chemical properties of lipids.

*Vice versa*, proteotoxicity-inducing agents can induce lipid accumulation and changes in lipid droplet size in various models, including UPR-ER-deficient, aged *C. elegans* [76], human hepatoma cell lines [118], and mice [119]. However, these studies employed gene expression and visible lipid-related morphological differences to study the effects of proteotoxicity. Attempts to study changes in lipid composition more directly have been made recently. Lipid profiling using ^1^H nuclear magnetic resonance (NMR) showed that protein misfolding caused by acyclic retinoid significantly reduced unsaturated FA content in a human cancer cell line [120]. Imaging by scanning electron microscopy and Raman spectroscopy of individual, tunicamycin-treated endothelial cells showed a decrease in ER phospholipid content [121]. Furthermore, mass-spectrometry-based lipid analysis showed that a short cultivation of *S. cerevisiae* in DTT is sufficient to induce substantial lipidomic changes, including an increase in overall PA and a shift of PA lipids toward a higher average acyl chain length and a greater unsaturation [122]. This occurred in an *IRE1*-independent manner, reinforcing the idea of a direct link between two types of parallel stresses, rather than one being a downstream response of the other.

## 8. Functional Genomic Approaches Identify New UPR-ER^LBS^ Components in *C. elegans*

In *C. elegans*, the functional mapping and characterization of genetic pathways is typically done by identifying mutants with a desired phenotype (e.g., activation of a reporter gene, synthetic lethality with another gene, increased sensitivity to a chemical, etc.). These classical genetic approaches have been extremely useful in pathway mapping and have helped identify novel lipid metabolism regulators of the UPR-ER [13,14]. For example, recent efforts have used functional genomics approaches to identify new players in the UPR-ER^LBS^ of *C. elegans*. Specifically, a reverse genetic screen depleting 1247 predicted metabolic genes by RNAi yielded 34 genes whose inactivation induces the UPR-ER, including previously known players in lipid homeostasis and new candidates whose specific links to UPR-ER have not been explored [13]. Another study screened 712 kinase and transcription factor genes and identified 8 genes whose inactivation suppresses the UPR-ER^LBS^ (induced by fat-6, mdt-15, or sams-1 RNAi) but not the UPR-ER^PT^ (induced by heat or tunicamycin) [14]. This is exciting as it provides a list of new genes that may specifically activate the UPR-ER^LBS^ or may selectively be required for a functional UPR-ER^LBS^, respectively. Collectively, these studies highlight the power of functional genomics in identifying novel inputs, components, or regulators of the UPR-ER^LBS^ pathway.

However, these approaches alone are not sufficient to delineate the full scope of UPR-ER activation and regulation and its interaction with lipid metabolism. These gaps can, however, be filled by other -omics-type approaches, including transcriptomics [17,18], proteomics, and, especially, metabolomics and lipidomics [17,71].

## 9. Metabolomics, Lipidomics, and Label-Free Imaging Are Powerful Emerging Tools to Gain Insights into UPR-ER^LBS^ Inputs and Outputs

The above noted genomic approaches have been successfully used in *C. elegans* to identify putative UPR-ER^LBS^ components, but many of these genes are not well understood. Exciting untapped potential to gain insights into the function of these genes is offered by the application of metabolomics and lipidomics technologies, as well as by computational modeling of metabolic networks composed of metabolites, corresponding enzymes, and their genes.

Metabolomics involves simultaneously quantifying the abundance of a large number of small molecules [123]. The estimated size of the complete *C. elegans* metabolome exceeds 10,000 molecules [124]. With advances in detection methods, the known metabolome in *C. elegans* now covers more than 1000 metabolites (excluding lipids) and is rapidly expanding [123]. Comparative metabolomic studies have already provided valuable insights into *C. elegans* aging, including the prediction of candidate biomarkers of aging [125].

Recently, non-conventional metabolomics and lipidomics approaches have been developed to reveal spatiotemporal insights at the whole-organismal level. Creative approaches like heteronuclear NMR live metabolomics in *C. elegans* allow temporal resolution of metabolite levels in live worms instead of at fixed time points [126], which may not reveal the effect of non-steady-state or time-dependent changes in metabolite levels on the UPR-ER. Moreover, although resolution barriers may currently prevent visualization of the ER, the novel use of coherent Raman spectroscopy imaging has allowed label-free and quantitative study of the tissue distribution of lipid-rich structures in live *C. elegans*, providing a direct way of visualizing lipid content in a spatially resolved manner [127]. The ability to accurately measure the fat content in different *C. elegans* tissue types with distinct UPR-ER patterns could be important for the accurate interpretation of the physiological importance of the UPR-ER^LBS^.

Attempting to integrate this wealth of genetic and metabolomic data, several metabolic network models have been developed for *C. elegans*, ultimately resulting in a consensus genome-scale metabolic network, termed WormJam [128,129,130]. Such organism-specific network reconstructions include all known metabolic reactions and the genes that encode each pertinent enzyme, thus providing a reference framework [123,131]. These networks can be combined with flux balance analysis, which is usually set to maximize biomass production [132] and given experimentally measured enzyme expression level constraints. This allows *in silico* predictions of how changes in the level of a metabolite or a mutation in a pertinent gene might affect the metabolomic landscape and, by extension, organismal parameters such as growth. This approach has been used to investigate aging in *C. elegans* [128], now with a metabolomics-integrated objective function tailored to the aging process [133]. Similarly, the combinatorial application of both targeted and untargeted metabolomics has been adopted in recent studies, yielding novel insights into the respective molecular mechanisms of the responses to toxins in human primary hepatocytes [134] and male mice [135].

Lipids play an essential role in biology, and over 40,000 types of lipids have been identified in humans [65]. Due to the diversity and abundance of lipids, lipidomics evolved from metabolomics as an independent branch, aiming to quantitatively determine the complete lipid composition in a sample. Lipidomics is mainly based on mass spectrometry (MS), which is more sensitive than NMR [11]. For details of different lipid analysis methods and their advantages and disadvantages in *C. elegans*, please see a recent review [11].

Currently, the major lipidomics database is LipidMaps [136]. However, unlike WormJam, the consensus *C. elegans* metabolomics model, no consensus *C. elegans* lipidome model currently exists [11].

## 10. Potential -omics Work Characterizing UPR-ER Inducing Metabolic Disturbances in *C. elegans*

**Figure 3:**
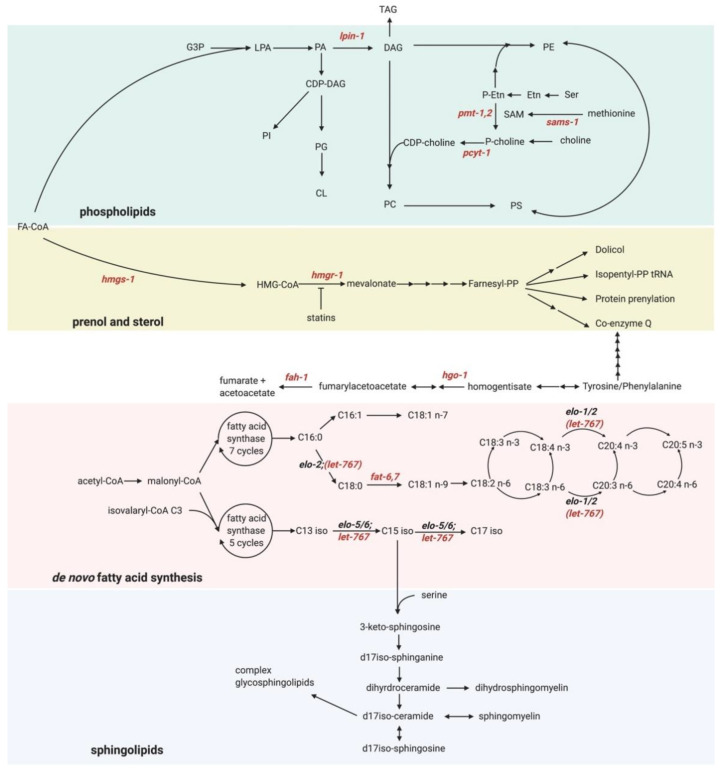
Overview of lipid synthesis pathways in *C. elegans* (adapted with permission from [143]). Genes colored in red are known to induce the UPR-ER when inactivated. Abbreviations: CDP-Cho: cytidine diphosphate choline; CDP-DAG: cytidine diphosphate diacylglycerol; CL: cardiolipin; CoA: coenzyme A; DAG: diacylglycerol; *elo-1/-2*: fatty acid elongation; Etn: ethanolamine; FA: fatty acid; *fah-1*: fumarylacetoacetate hydrolase; *fat-6/-7*: fatty acid desaturase 6/7; G3P: glucose-3 phosphate; *hgo-1*: homogentisate 1,2-dioxygenase; HMG-CoA: 3-hydroxy-3-methyl-glutaryl-coenzyme A; *hmgr-1*: hydroxymethylglutaryl-CoA reductase; *hmgs-1*: hydroxymethylglutaryl-CoA synthase; LPA: lysophosphatidic acid; *lpin-1*: lipin (mammalian lipodystrophy associated) homolog; PA: phosphatidic acid; PC: phosphatidylcholine; PE: phosphatidylethanolamine; P-Etn: phosphoethanolamine; PG: phosphatidylglycerol; PI: phosphatidylinositol; *pmt-2*: phosphoethanolamine methyltransferase; PP: pyrophosphate; PS: phosphatidylserine; Ser: serine; SAM: S-adenosyl methionine; *sams-1*: S-adenosyl methionine synthetase; TAG: triacylglycerol. (Created with BioRender.com, Toronto, ON, Canada).

We now discuss the application of these exciting methods and tools to better understand some of the putative new UPR-ER activators. Our functional genomic screen identified several genes that activate the UPR-ER [13]. Consistent with previous studies, we identified genes that induce proteotoxicity-independent UPR-ER^LBS^, such as the FA desaturases fat-6 and fat-7 [71]; the PC synthesis enzymes pcyt-1 and sams-1 [137]; lpin-1, which is linked to the synthesis of ω-6 PUFA-containing phospholipids [138,139,140]; and the mevalonate pathway components hmgs-1 and hmgr-1 [141,142] (Figure 3). We also found several other metabolic genes whose inactivation may activate the UPR-ER^LBS^, but via unclear mechanisms. Omics-type methods such as those outlined above could help gain insight into how inactivation of these genes activates the UPR-ER^LBS^.

For example, nmt-1 encodes N-myristoyl transferase, which irreversibly attaches a myristate (C14 FA) moiety to the N-terminal glycine of proteins that participate in signal transduction. A global N-myristoylated proteome consisting of >100 proteins in human cells has been determined by quantitative proteomics studies [144]. Intriguingly, like in *C. elegans*, chemical inhibition of N-myristoyl transferase activity in human cell lines upregulates proteins involved in ER stress [145], suggesting that myristoylation may be required for ER homeostasis via conserved protein targets. Identifying targets of *C. elegans* NMT-1 using comparative proteomics followed by genetic validation studies may pinpoint NMT-1 downstream targets whose myristoylation is involved in maintaining ER homeostasis.

Another interesting gene is hgo-1, which encodes for homogentisate 1,2-dioxygenase; HGO-1 breaks down aromatic amino acids (tyrosine and phenylalanine; Figure 3). hgo-1 loss not only activates the UPR-ER, but also results in increased oxidative stress [146]. Moreover, inactivation of the fumarylacetoacetate hydrolase fah-1, an enzyme downstream of hgo-1 in the tyrosine/phenylamine metabolism pathway, also causes UPR-ER induction (Figure 3). This suggests that this breakdown pathway may be essential to preventing ER stress. fah-1 RNAi results in growth defects and hsp-4 upregulation in *C. elegans* due to toxic upstream metabolite buildup, and the growth defect is suppressed in fah-1/hgo-1 double RNAi treated worms [147]. However, hgo-1 RNAi also induces hsp-4 expression, suggesting that the growth defect can be uncoupled from UPR-ER activation in tyrosine/phenylalanine metabolism pathway mutants. Remarkably, inhibition of phenylalanine hydroxylase, the initial enzyme in the aromatic amino acid catabolism pathway, has been implicated in changing FA composition, which cannot be rescued by tyrosine supplementation [148,149]. Collectively, these studies point to phenylalanine build-up as a potential cause for UPR-ER activation, consistent with previous reports that phenylalanine increases membrane permeability by insertion into the membrane in liposomes [150]. However, whether phenylalanine induces the UPR-ER via alterations in membrane properties in vivo has not been tested. Untargeted or semi-targeted lipidomics profiling could reveal if and how this phenylalanine catabolism pathway induces the UPR-ER.

Finally, let-767, whose inactivation also induces the UPR-ER and causes developmental arrest, is a 3-ketoacyl-CoA reductase localized to the ER. let-767 is necessary to sysnthesize long-chain and mono-methyl branched-chain FAs [151] (Figure 3), both important precursors for sphingolipid synthesis in *C. elegans* [151,152]. Yet, how let-767 maintains ER homeostasis is unknown. LET-767 possesses steroid-modifying activity in worms [153], but this function is dispensable for normal development [151]. Gas chromatography mass spectrometry (GC-MS) analysis of FA profiles revealed that let-767 RNAi caused a decrease in C15iso and C17iso monomethyl branched-chain FAs and sphingolipids [151,152,154]. Consistently, iso-15:0, iso-17:0, and iso-19:0 monomethyl branched-chain FAs are sufficient to rescue the developmental arrest due to let-767 RNAi [151]. Interestingly, a recent study showed that let-767 RNAi results in severely disturbed ER morphology, which can be rescued by supplementation with wildtype worm lysate, but not by supplementation with mono-methyl branched chain fatty acid [155]. This suggests that LET-767 disruption induces ER stress through an unknown mechanism, independently of branched-chain FA synthesis [156]. Perhaps defective synthesis of long-chain FAs and/or sphingolipids, both of which have been linked to UPR-ER regulation [157], is the culprit. Indeed, very-long-chain FAs (>20C) can increase membrane saturation [158], which activates the UPR-ER^LBS^ [71]. Targeted lipidomics in let-767-depleted worms would be a powerful approach to quantify changes in different very-long-chain FA levels in sphingolipids, possibly after ER membrane extraction [159].

## 11. Conclusions

Although the role of the UPR-ER in maintaining a healthy proteome is well understood, recent research has highlighted bidirectional feedback between lipid metabolism and the UPR-ER in several models, including *C. elegans*. Recent progress has aimed at distinguishing proteotoxicity-induced UPR-ER^PT^ from lipotoxicity-induced UPR-ER^LBS^, yielding valuable insights into the LBS-induced activation mechanism of IRE-1 and the genetic regulation of membrane lipid homeostasis at a molecular level. Despite clear differences in proteotoxicity and lipotoxicity in terms of the activation mechanism of IRE-1 and transcriptional outputs, the two stresses nonetheless crosstalk and likely contribute to synergistic UPR-ER activation. Although lipotoxicity is now established as an ER stress inducer parallel to and independent of proteotoxicity, our understanding of how, molecularly, specific lipid metabolism pathway defects induce the UPR-ER remains limited. Similarly, we are only beginning to understand how the lipidome is influenced by the UPR-ER in response to a particular insult. We anticipate that using -omics tools, particularly metabolomics and lipidomics, will lead to new findings and more comprehensive answers to such questions in the future.

## References

[1] Senft D., Ronai Z.A. (2015). UPR, autophagy, and mitochondria crosstalk underlies the ER stress response. Trends Biochem. Sci..

[2] Adams C.J., Kopp M.C., Larburu N., Nowak P.R., Ali M.M.U. (2019). Structure and Molecular Mechanism of ER Stress Signaling by the Unfolded Protein Response Signal Activator IRE1. Front. Mol. Biosci..

[3] Oakes S.A., Papa F.R. (2015). The Role of Endoplasmic Reticulum Stress in Human Pathology. Annu. Rev. Pathol. Mech. Dis..

[4] Hetz C., Papa F.R. (2018). The Unfolded Protein Response and Cell Fate Control. Mol. Cell.

[5] Lindholm D., Korhonen L., Eriksson O., Kõks S. (2017). Recent Insights into the Role of Unfolded Protein Response in ER Stress in Health and Disease. Front. Cell Dev. Biol..

[6] McQuiston A., Diehl J.A. (2017). Recent insights into PERK-dependent signaling from the stressed endoplasmic reticulum. F1000Research.

[7] Hillary R.F., Fitzgerald U. (2018). A lifetime of stress: ATF6 in development and homeostasis. J. Biomed. Sci..

[8] Walter P., Ron D. (2011). The Unfolded Protein Response: From Stress Pathway to Homeostatic Regulation. Science.

[9] Lai C.-H., Chou C.-Y., Ch’Ang L.-Y., Liu C.-S., Lin W.-C. (2000). Identification of Novel Human Genes Evolutionarily Conserved in Caenorhabditis elegans by Comparative Proteomics. Genome Res..

[10] Kaletta T., Hengartner M.O. (2006). Finding function in novel targets: *C. elegans* as a model organism. Nat. Rev. Drug Discov..

[11] Witting M., Schmitt-Kopplin P. (2016). The Caenorhabditis elegans lipidome. Arch. Biochem. Biophys..

[12] Zhang Y., Zou X., Ding Y., Wang H., Wu X., Liang B. (2013). Comparative genomics and functional study of lipid metabolic genes in Caenorhabditis elegans. BMC Genom..

[13] Ho N., Yap W.S., Xu J., Wu H., Koh J.H., Bin Goh W.W., George B., Chong S.C., Taubert S., Thibault G. (2020). Stress sensor Ire1 deploys a divergent transcriptional program in response to lipid bilayer stress. J. Cell Biol..

[14] Venz R., Korosteleva A., Jongsma E., Ewald C.Y. (2020). Combining Auxin-Induced Degradation and RNAi Screening Identifies Novel Genes Involved in Lipid Bilayer Stress Sensing in Caenorhabditis elegans. G3 Genes Genomes Genet..

[15] Singh J., Aballay A. (2017). Endoplasmic Reticulum Stress Caused by Lipoprotein Accumulation Suppresses Immunity against Bacterial Pathogens and Contributes to Immunosenescence. mBio.

[16] Marza E., Taouji S., Barroso K., Raymond A.-A., Guignard L., Bonneu M., Pallares-Lupon N., Dupuy J.-W., Fernandez-Zapico M.E., Rosenbaum J. (2015). Genome-wide screen identifies a novel p97/CDC -48-dependent pathway regulating ER -stress-induced gene transcription. EMBO Rep..

[17] Koh J.H., Wang L., Beaudoin-Chabot C., Thibault G. (2018). Lipid bilayer stress-activated IRE-1 modulates autophagy during endoplasmic reticulum stress. J. Cell Sci..

[18] Shen X., Ellis R.E., Sakaki K., Kaufman R.J. (2005). Genetic Interactions Due to Constitutive and Inducible Gene Regulation Mediated by the Unfolded Protein Response in *C. elegans*. PLoS Genet..

[19] Taylor R.C., Dillin A. (2013). XBP-1 Is a Cell-Nonautonomous Regulator of Stress Resistance and Longevity. Cell.

[20] Imanikia S., Sheng M., Castro C., Griffin J.L., Taylor R.C. (2019). XBP-1 Remodels Lipid Metabolism to Extend Longevity. Cell Rep..

[21] Özbey N.P., Imanikia S., Krueger C., Hardege I., Morud J., Sheng M., Schafer W.R., Casanueva M.O., Taylor R.C. (2020). Tyramine Acts Downstream of Neuronal XBP-1s to Coordinate Inter-tissue UPRER Activation and Behavior in *C. elegans*. Dev. Cell.

[22] Daniele J.R., Higuchi-Sanabria R., Durieux J., Monshietehadi S., Ramachandran V., Tronnes S.U., Kelet N., Sanchez M., Metcalf M.G., Garcia G. (2020). UPRER promotes lipophagy independent of chaperones to extend life span. Sci. Adv..

[23] Nikawa J.-I., Yamashita S. (1992). IRE1 encodes a putative protein kinase containing a membrane-spanning domain and is required for inositol phototrophy in Saccharomyces cerevisiae. Mol. Microbiol..

[24] Cox J.S., Shamu C.E., Walter P. (1993). Transcriptional induction of genes encoding endoplasmic reticulum resident proteins requires a transmembrane protein kinase. Cell.

[25] Morl K., Ma W., Gething M.-J., Sambrook J. (1993). A transmembrane protein with a cdc2+CDC28-related kinase activity is required for signaling from the ER to the nucleus. Cell.

[26] Carrara M., Prischi F., Nowak P.R., Ali M.M.U. (2015). Crystal structures reveal transient PERK luminal domain tetramerization in endoplasmic reticulum stress signaling. EMBO J..

[27] Lu Y., Liang F.-X., Wang X. (2014). A Synthetic Biology Approach Identifies the Mammalian UPR RNA Ligase RtcB. Mol. Cell.

[28] Calfon M., Zeng H., Urano F., Till J.H., Hubbard S.R., Harding H.P., Clark S.G., Ron D. (2002). IRE1 Couples Endoplasmic Reticulum Load to Secretory Capacity by Processing the XBP-1 MRNA. Nature.

[29] Yoshida H., Matsui T., Yamamoto A., Okada T., Mori K. (2001). XBP1 mRNA Is Induced by ATF6 and Spliced by IRE1 in Response to ER Stress to Produce a Highly Active Transcription Factor. Cell.

[30] Travers K.J., Patil C.K., Wodicka L., Lockhart D.J., Weissman J.S., Walter P. (2000). Functional and Genomic Analyses Reveal an Essential Coordination between the Unfolded Protein Response and ER-Associated Degradation. Cell.

[31] Hetz C., Glimcher L.H. (2009). Fine-Tuning of the Unfolded Protein Response: Assembling the IRE1α Interactome. Mol. Cell.

[32] Hollien J., Weissman J.S. (2006). Decay of Endoplasmic Reticulum-Localized mRNAs During the Unfolded Protein Response. Science.

[33] Cross B.C.S., Bond P.J., Sadowski P.G., Jha B.K., Zak J., Goodman J.M., Silverman R.H., Neubert T.A., Baxendale I.R., Ron D. (2012). The molecular basis for selective inhibition of unconventional mRNA splicing by an IRE1-binding small molecule. Proc. Natl. Acad. Sci. USA.

[34] Kimmig P., Diaz M., Zheng J., Williams C.C., Lang A., Aragón T., Li H., Walter P. (2012). The unfolded protein response in fission yeast modulates stability of select mRNAs to maintain protein homeostasis. eLife.

[35] Ho N., Xu C., Thibault G. (2018). From the unfolded protein response to metabolic diseases—Lipids under the spotlight. J. Cell Sci..

[36] Smith M.H., Ploegh H.L., Weissman J.S. (2011). Road to Ruin: Targeting Proteins for Degradation in the Endoplasmic Reticulum. Science.

[37] Struwe W.B., Hughes B.L., Osborn D.W., Boudreau E.D., Shaw K.M.D., Warren C.E. (2009). Modeling a congenital disorder of glycosylation type I in *C. elegans*: A genome-wide RNAi screen for N-glycosylation-dependent loci. Glycobiology.

[38] Richardson C.E., Kooistra T.G., Kim D.H. (2010). An essential role for XBP-1 in host protection against immune activation in *C. elegans*. Nat. Cell Biol..

[39] Salzberg Y., Coleman A.J., Celestrin K., Cohen-Berkman M., Biederer T., Henis-Korenblit S., Bülow H.E. (2017). Reduced Insulin/Insulin-Like Growth Factor Receptor Signaling Mitigates Defective Dendrite Morphogenesis in Mutants of the ER Stress Sensor IRE-1. PLoS Genet..

[40] Ryoo H.D., Domingos P.M., Kang M.-J., Steller H. (2006). Unfolded protein response in a Drosophila model for retinal degeneration. EMBO J..

[41] Reimold A.M., Etkin A., Clauss I., Perkins A., Friend D.S., Zhang J., Horton H.F., Scott A., Orkin S.H., Byrne M.C. (2000). An essential role in liver development for transcription factor XBP-1. Genome Res..

[42] Bertolotti A., Zhang Y., Hendershot L.M., Harding H.P., Ron D. (2000). Dynamic interaction of BiP and ER stress transducers in the unfolded-protein response. Nat. Cell Biol..

[43] Novoa I., Zeng H., Harding H.P., Ron D. (2001). Feedback Inhibition of the Unfolded Protein Response by GADD34-Mediated Dephosphorylation of eIF2α. J. Cell Biol..

[44] Rousakis A., Vlassis A., Vlanti A., Patera S., Thireos G., Syntichaki P. (2013). The general control nonderepressible-2 kinase mediates stress response and longevity induced by target of rapamycin inactivation inCaenorhabditis elegans. Aging Cell.

[45] Statzer C., Venz R., Bland M., Robida-Stubbs S., Meng J., Patel K., Emsley R., Petrovic D., Liu P., Morantte I. (2020). ATF-4 and Hydrogen Sulfide Signalling Mediate Longevity from Inhibition of Translation or MTORC1. bioRxiv.

[46] Shen X., Ellis R.E., Lee K., Liu C.-Y., Yang K., Solomon A., Yoshida H., Morimoto R., Kurnit D.M., Mori K. (2001). Complementary Signaling Pathways Regulate the Unfolded Protein Response and Are Required for *C. elegans* Development. Cell.

[47] Harding H.P., Zhang Y., Bertolotti A., Zeng H., Ron D. (2000). Perk Is Essential for Translational Regulation and Cell Survival during the Unfolded Protein Response. Mol. Cell.

[48] Harding H.P., Zeng H., Zhang Y., Jungries R., Chung P., Plesken H., Sabatini D.D., Ron D. (2001). Diabetes Mellitus and Exocrine Pancreatic Dysfunction in Perk −/− Mice Reveals a Role for Translational Control in Secretory Cell Survival. Mol. Cell.

[49] Delépine M., Nicolino M., Barrett T.G., Golamaully M., Lathrop G.M., Julier C. (2000). EIF2AK3, encoding translation initiation factor 2-α kinase 3, is mutated in patients with Wolcott-Rallison syndrome. Nat. Genet..

[50] Lee K., Tirasophon W., Shen X., Michalak M., Prywes R., Okada T., Yoshida H., Mori K., Kaufman R.J. (2002). IRE1-mediated unconventional mRNA splicing and S2P-mediated ATF6 cleavage merge to regulate XBP1 in signaling the unfolded protein response. Genes Dev..

[51] Yamamoto K., Sato T., Matsui T., Sato M., Okada T., Yoshida H., Harada A., Mori K. (2007). Transcriptional Induction of Mammalian ER Quality Control Proteins Is Mediated by Single or Combined Action of ATF6α and XBP1. Dev. Cell.

[52] Shoulders M.D., Ryno L.M., Genereux J.C., Moresco J.J., Tu P.G., Wu C., Yates J.R., Su A.I., Kelly J.W., Wiseman R.L. (2013). Stress-Independent Activation of XBP1s and/or ATF6 Reveals Three Functionally Diverse ER Proteostasis Environments. Cell Rep..

[53] Waldherr S.M., Strovas T.J., Vadset T.A., Liachko N.F., Kraemer B.C. (2019). Constitutive XBP-1s-mediated activation of the endoplasmic reticulum unfolded protein response protects against pathological tau. Nat. Commun..

[54] Wu J., Rutkowski D.T., Dubois M., Swathirajan J., Saunders T., Wang J., Song B., Yau G.D.-Y., Kaufman R.J. (2007). ATF6α Optimizes Long-Term Endoplasmic Reticulum Function to Protect Cells from Chronic Stress. Dev. Cell.

[55] Wang S., Hu B., Ding Z., Dang Y., Wu J., Li D., Liu X., Xiao B., Zhang W., Ren R. (2018). ATF6 safeguards organelle homeostasis and cellular aging in human mesenchymal stem cells. Cell Discov..

[56] Kohl S., Zobor D., Chiang W.-C., Weisschuh N., Staller J., Menendez I.G., Chang S., Beck S.C., Garrido M.G., Sothilingam V. (2015). Mutations in the unfolded protein response regulator ATF6 cause the cone dysfunction disorder achromatopsia. Nat. Genet..

[57] Havel R.J., Eder H.A., Bragdon J.H. (1955). The Distribution and Chemical Composition of Ultracentrifugally Separated Lipoproteins in Human Serum. J. Clin. Investig..

[58] Fahy E., Subramaniam S., Brown H.A., Glass C.K., Merrill A.H., Murphy R.C., Raetz C.R.H., Russell D.W., Seyama Y., Shaw W. (2005). A comprehensive classification system for lipids. J. Lipid Res..

[59] Haucke V., Di Paolo G. (2007). Lipids and lipid modifications in the regulation of membrane traffic. Curr. Opin. Cell Biol..

[60] Parks L.W., Casey W.M. (1995). Physiological Implications of Sterol Biosynthesis in Yeast. Annu. Rev. Microbiol..

[61] Van Meer G., De Kroon A.I.P.M. (2010). Lipid map of the mammalian cell. J. Cell Sci..

[62] Vance J.E. (2014). Phospholipid Synthesis and Transport in Mammalian Cells. Traffic.

[63] Potting C., Tatsuta T., König T., Haag M., Wai T., Aaltonen M.J., Langer T. (2013). TRIAP1/PRELI Complexes Prevent Apoptosis by Mediating Intramitochondrial Transport of Phosphatidic Acid. Cell Metab..

[64] Mesmin B. (2016). Mitochondrial lipid transport and biosynthesis: A complex balance. J. Cell Biol..

[65] Wang R., Li B., Lam S.-M., Shui G. (2020). Integration of lipidomics and metabolomics for in-depth understanding of cellular mechanism and disease progression. J. Genet. Genom..

[66] Ariyama H., Kono N., Matsuda S., Inoue T., Arai H. (2010). Decrease in Membrane Phospholipid Unsaturation Induces Unfolded Protein Response. J. Biol. Chem..

[67] Pineau L., Ferreira T. (2010). Lipid-induced ER stress in yeast and β cells: Parallel trails to a common fate. FEMS Yeast Res..

[68] Pineau L., Colas J., Dupont S., Beney L., Fleurat-Lessard P., Berjeaud J.-M., Bergès T., Ferreira T. (2009). Lipid-Induced ER Stress: Synergistic Effects of Sterols and Saturated Fatty Acids. Traffic.

[69] Volmer R., Van Der Ploeg K., Ron D. (2013). Membrane lipid saturation activates endoplasmic reticulum unfolded protein response transducers through their transmembrane domains. Proc. Natl. Acad. Sci. USA.

[70] Sommerweiss D., Gorski T., Richter S., Garten A., Kiess W. (2013). Oleate rescues INS-1E β-cells from palmitate-induced apoptosis by preventing activation of the unfolded protein response. Biochem. Biophys. Res. Commun..

[71] Hou N.S., Gutschmidt A., Choi D.Y., Pather K., Shi X., Watts J.L., Hoppe T., Taubert S. (2014). Activation of the endoplasmic reticulum unfolded protein response by lipid disequilibrium without disturbed proteostasis in vivo. Proc. Natl. Acad. Sci. USA.

[72] Thibault G., Shui G., Kim W., McAlister G.C., Ismail N., Gygi S.P., Wenk M.R., Ng D.T. (2012). The Membrane Stress Response Buffers Lethal Effects of Lipid Disequilibrium by Reprogramming the Protein Homeostasis Network. Mol. Cell.

[73] Fu S., Yang L., Li P., Hofmann O., Dicker L., Hide W., Lin X., Watkins S.M., Ivanov A.R., Hotamisligil G.S. (2011). Aberrant lipid metabolism disrupts calcium homeostasis causing liver endoplasmic reticulum stress in obesity. Nat. Cell Biol..

[74] Jo H., Shim J., Lee J.H., Lee J., Kim J.B. (2009). IRE-1 and HSP-4 Contribute to Energy Homeostasis via Fasting-Induced Lipases in *C. elegans*. Cell Metab..

[75] Koh J.H., Wang L., Beaudoin-Chabot C., Thibault G. (2018). Lipid Perturbation-Activated IRE-1 Modulates Autophagy and Lipolysis during Endoplasmic Reticulum Stress. bioRxiv.

[76] Kyriakakis E., Charmpilas N., Tavernarakis N. (2017). Differential adiponectin signalling couples ER stress with lipid metabolism to modulate ageing in *C. elegans*. Sci. Rep..

[77] Huang H.-W., Zeng X., Rhim T., Ron D., Ryoo H.D. (2017). The requirement of IRE1 and XBP1 in resolving physiological stress during Drosophila development. J. Cell Sci..

[78] Harayama T., Riezman H. (2018). Understanding the diversity of membrane lipid composition. Nat. Rev. Mol. Cell Biol..

[79] Wortmann S.B., Espeel M., Almeida L., Reimer A., Bosboom D., Roels F., De Brouwer A.P., Wevers R.A. (2014). Inborn errors of metabolism in the biosynthesis and remodelling of phospholipids. J. Inherit. Metab. Dis..

[80] Liu X., Strable M.S., Ntambi J.M. (2011). Stearoyl CoA Desaturase 1: Role in Cellular Inflammation and Stress. Adv. Nutr..

[81] Dobrzyń A. (2004). The Role of Stearoyl-CoA Desaturase in Body Weight Regulation. Trends Cardiovasc. Med..

[82] Matsui H., Yokoyama T., Sekiguchi K., Iijima D., Sunaga H., Maniwa M., Ueno M., Iso T., Arai M., Kurabayashi M. (2012). Stearoyl-CoA Desaturase-1 (SCD1) Augments Saturated Fatty Acid-Induced Lipid Accumulation and Inhibits Apoptosis in Cardiac Myocytes. PLoS ONE.

[83] Mauvoisin D., Mounier C. (2011). Hormonal and nutritional regulation of SCD1 gene expression. Biochimie.

[84] Tian L., Shao W., Ip W., Song Z., Badakhshi Y., Jin T. (2019). The developmental Wnt signaling pathway effector β-catenin/TCF mediates hepatic functions of the sex hormone estradiol in regulating lipid metabolism. PLoS Biol..

[85] Samuel W., Nagineni C.N., Kutty R.K., Parks W.T., Gordon J.S., Prouty S.M., Hooks J.J., Wiggert B. (2002). Transforming Growth Factor-β Regulates Stearoyl Coenzyme A Desaturase Expression through a Smad Signaling Pathway. J. Biol. Chem..

[86] Samuel W., Kutty R.K., Nagineni S., Gordon J.S., Prouty S.M., Chandraratna R.A.S., Wiggert B. (2001). Regulation of Stearoyl Coenzyme A Desaturase Expression in Human Retinal Pigment Epithelial Cells by Retinoic Acid. J. Biol. Chem..

[87] Mori H., Dugan C.E., Nishii A., Benchamana A., Li Z., Cadenhead T.S., Das A.K., Evans C.R., Overmyer K.A., Romanelli S.M. (2020). The Molecular and Metabolic Program for Adaptation of White Adipocytes to Cool Physiologic Temperatures. bioRxiv.

[88] Chen M., Huang J. (2019). The expanded role of fatty acid metabolism in cancer: New aspects and targets. Precis. Clin. Med..

[89] Cheng M., Bhujwalla Z.M., Glunde K. (2016). Targeting Phospholipid Metabolism in Cancer. Front. Oncol..

[90] Sharma B., Kanwar S.S. (2018). Phosphatidylserine: A cancer cell targeting biomarker. Semin. Cancer Biol..

[91] Huang J., Fan X.-X., He J., Pan H., Liyan H., Huang L., Jiang Z., Yao X.-J., Liu L., Leung E.L.-H. (2016). SCD1 is associated with tumor promotion, late stage and poor survival in lung adenocarcinoma. Oncotarget.

[92] Ackerman D., Simon M.C. (2014). Hypoxia, lipids, and cancer: Surviving the harsh tumor microenvironment. Trends Cell Biol..

[93] Munir R., Lisec J., Swinnen J.V., Zaidi N. (2019). Lipid metabolism in cancer cells under metabolic stress. Br. J. Cancer.

[94] Oakes S.A. (2020). Endoplasmic Reticulum Stress Signaling in Cancer Cells. Am. J. Pathol..

[95] Mahameed M., Boukeileh S., Obiedat A., Darawshi O., Dipta P., Rimon A., McLennan G., Fassler R., Reichmann D., Karni R. (2020). Pharmacological induction of selective endoplasmic reticulum retention as a strategy for cancer therapy. Nat. Commun..

[96] Raymundo D.P., Doultsinos D., Guillory X., Carlesso A., Eriksson L.A., Chevet E. (2020). Pharmacological Targeting of IRE1 in Cancer. Trends Cancer.

[97] Pagliassotti M.J., Kim P.Y., Estrada A.L., Stewart C.M., Gentile C.L. (2016). Endoplasmic reticulum stress in obesity and obesity-related disorders: An expanded view. Metabolism.

[98] Basseri S., Austin R.C. (2011). Endoplasmic Reticulum Stress and Lipid Metabolism: Mechanisms and Therapeutic Potential. Biochem. Res. Int..

[99] Gregor M.F., Yang L., Fabbrini E., Mohammed B.S., Eagon J.C., Hotamisligil G.S., Klein S. (2008). Endoplasmic Reticulum Stress Is Reduced in Tissues of Obese Subjects After Weight Loss. Diabetes.

[100] Sohn M., Ivanova P., Brown H.A., Toth D.J., Varnai P., Kim Y.J., Balla T. (2016). Lenz-Majewski mutations in PTDSS1 affect phosphatidylinositol 4-phosphate metabolism at ER-PM and ER-Golgi junctions. Proc. Natl. Acad. Sci. USA.

[101] Wu H., Carvalho P., Voeltz G.K. (2018). Here, there, and everywhere: The importance of ER membrane contact sites. Science.

[102] Funato K., Riezman H., Muñiz M. (2020). Vesicular and non-vesicular lipid export from the ER to the secretory pathway. Biochim. Biophys. Acta Mol. Cell Biol. Lipids.

[103] Ozcan U., Yilmaz E., Özcan L., Furuhashi M., Vaillancourt E., Smith R.O., Görgün C.Z., Hotamisligil G.S. (2006). Chemical Chaperones Reduce ER Stress and Restore Glucose Homeostasis in a Mouse Model of Type 2 Diabetes. Science.

[104] Mai C.T., Le Q.G., Ishiwata-Kimata Y., Takagi H., Kohno K., Kimata Y. (2018). 4-Phenylbutyrate suppresses the unfolded protein response without restoring protein folding in Saccharomyces cerevisiae. FEMS Yeast Res..

[105] Nissar A.U., Sharma L., Mudasir M.A., Nazir L.A., Umar S.A., Sharma P.R., Vishwakarma R.A., Tasduq S.A. (2017). Chemical chaperone 4-phenyl butyric acid (4-PBA) reduces hepatocellular lipid accumulation and lipotoxicity through induction of autophagy. J. Lipid Res..

[106] Arai Y., Choi B., Kim B.J., Rim W., Park S., Park H., Ahn J., Park H. (2019). Tauroursodeoxycholic acid (TUDCA) counters osteoarthritis by regulating intracellular cholesterol levels and membrane fluidity of degenerated chondrocytes. Biomater. Sci..

[107] Lee H., Lee G., Kim H., Lee Y., Chae H. (2020). Phosphatidylinositol 3-kinase-δ controls endoplasmic reticulum membrane fluidity and permeability in fungus-induced allergic inflammation in mice. Br. J. Pharmacol..

[108] Wei Y., Wang D., Gentile C.L., Pagliassotti M.J. (2009). Reduced endoplasmic reticulum luminal calcium links saturated fatty acid-mediated endoplasmic reticulum stress and cell death in liver cells. Mol. Cell. Biochem..

[109] Bi J., Wang W., Liu Z., Huang X., Jiang Q., Liu G., Wang Y., Huang X. (2014). Seipin Promotes Adipose Tissue Fat Storage through the ER Ca^2+^-ATPase SERCA. Cell Metab..

[110] Kitai Y., Ariyama H., Kono N., Oikawa D., Iwawaki T., Arai H. (2013). Membrane lipid saturation activates IRE1α without inducing clustering. Genes Cells.

[111] Promlek T., Ishiwata-Kimata Y., Shido M., Sakuramoto M., Kohno K., Kimata Y. (2011). Membrane aberrancy and unfolded proteins activate the endoplasmic reticulum stress sensor Ire1 in different ways. Mol. Biol. Cell.

[112] Deguil J., Pineau L., Snyder E.C.R., Dupont S., Beney L., Gil A., Frapper G., Ferreira T. (2011). Modulation of Lipid-Induced ER Stress by Fatty Acid Shape. Traffic.

[113] Shyu P., Ng B.S.H., Ho N., Chaw R., Seah Y.L., Marvalim C., Thibault G. (2019). Membrane phospholipid alteration causes chronic ER stress through early degradation of homeostatic ER-resident proteins. Sci. Rep..

[114] Boslem E., Weir J.M., MacIntosh G., Sue N., Cantley J., Meikle P.J., Biden T.J. (2013). Alteration of Endoplasmic Reticulum Lipid Rafts Contributes to Lipotoxicity in Pancreatic β-Cells. J. Biol. Chem..

[115] Sharpe H.J., Stevens T.J., Munro S. (2010). A Comprehensive Comparison of Transmembrane Domains Reveals Organelle-Specific Properties. Cell.

[116] Singh S., Trikha S., Bhowmick D.C., Sarkar A.A., Jeremic A.M. (2015). Role of Cholesterol and Phospholipids in Amylin Misfolding, Aggregation and Etiology of Islet Amyloidosis. Cannabinoids Neuropsychiatr. Disord..

[117] Bogdanov M., Dowhan W. (1998). Phospholipid-assisted protein folding: Phosphatidylethanolamine is required at a late step of the conformational maturation of the polytopic membrane protein lactose permease. EMBO J..

[118] Lee J.-S., Mendez R., Heng H.H., Yang Z.-Q., Zhang K. (2012). Pharmacological ER stress promotes hepatic lipogenesis and lipid droplet formation. Am. J. Transl. Res..

[119] Kim S.H., Kwon D.-Y., Kwak J.-H., Lee S., Lee Y.-H., Yun J., Son T.G., Jung Y.-S. (2018). Tunicamycin-Induced ER Stress is Accompanied with Oxidative Stress via Abrogation of Sulfur Amino Acids Metabolism in the Liver. Int. J. Mol. Sci..

[120] Qin X., Su T., Yu W., Kojima S. (2020). Lipid desaturation-associated endoplasmic reticulum stress regulates MYCN gene expression in hepatocellular carcinoma cells. Cell Death Dis..

[121] Bik E., Mielniczek N., Jarosz M., Denbigh J., Budzynska R., Baranska M., Majzner K. (2019). Tunicamycin induced endoplasmic reticulum changes in endothelial cells investigated in vitro by confocal Raman imaging. Analyst.

[122] Reinhard J., Mattes C., Väth K., Radanović T., Surma M.A., Klose C., Ernst R. (2020). A Quantitative Analysis of Cellular Lipid Compositions During Acute Proteotoxic ER Stress Reveals Specificity in the Production of Asymmetric Lipids. Front. Cell Dev. Biol..

[123] Witting M., Hastings J., Rodriguez N., Joshi C.J., Hattwell J.P.N., Ebert P.R., Van Weeghel M., Gao A.W., Wakelam M.J.O., Houtkooper R.H. (2018). Modeling Meets Metabolomics—The WormJam Consensus Model as Basis for Metabolic Studies in the Model Organism Caenorhabditis elegans. Front. Mol. Biosci..

[124] Artyukhin A.B., Zhang Y.K., Akagi A.E., Panda O., Sternberg P.W., Schroeder F.C. (2018). Metabolomic “Dark Matter” Dependent on Peroxisomal β-Oxidation in Caenorhabditis elegans. J. Am. Chem. Soc..

[125] Sharma R., Ramanathan A. (2020). The Aging Metabolome—Biomarkers to Hub Metabolites. Proteomics.

[126] Nguyen T.T.M., An Y.J., Cha J.W., Ko Y.-J., Lee H., Chung C.H., Jeon S.-M., Lee J., Park S. (2020). Real-Time In-Organism NMR Metabolomics Reveals Different Roles of AMP-Activated Protein Kinase Catalytic Subunits. Anal. Chem..

[127] Chen W.-W., Lemieux G.A., Camp C.H., Chang T.-C., Ashrafi K., Cicerone M.T. (2020). Spectroscopic coherent Raman imaging of Caenorhabditis elegans reveals lipid particle diversity. Nat. Chem. Biol..

[128] Gebauer J., Gentsch C., Mansfeld J., Schmeißer K., Waschina S., Brandes S., Klimmasch L., Zamboni N., Zarse K., Schuster S. (2016). A Genome-Scale Database and Reconstruction of Caenorhabditis elegans Metabolism. Cell Syst..

[129] Yilmaz L.S., Walhout A.J.M. (2016). A Caenorhabditis elegans Genome-Scale Metabolic Network Model. Cell Syst..

[130] Ma L., Chan A.H.C., Hattwell J., Ebert P., Schirra H.J. (2017). Systems Biology Analysis Using a Genome-Scale Metabolic Model Shows That Phosphine Triggers Global Metabolic Suppression in a Resistant Strain of C. Elegans. bioRxiv.

[131] Hastings J., Mains A., Artal-Sanz M., Bergmann S., Braeckman B.P., Bundy J.G., Cabreiro F., Dobson P., Ebert P., Hattwell J. (2017). WormJam: A consensus *C. elegans* Metabolic Reconstruction and Metabolomics Community and Workshop Series. Worm.

[132] Orth J.D., Thiele I., Palsson B.O. (2010). What is flux balance analysis?. Nat. Biotechnol..

[133] Hastings J., Mains A., Virk B., Rodriguez N., Murdoch S., Pearce J., Bergmann S., Le Novère N., Casanueva O. (2019). Multi-Omics and Genome-Scale Modeling Reveal a Metabolic Shift During *C. elegans* Aging. Front. Mol. Biosci..

[134] Xu L., Zhao Q., Luo J., Ma W., Jin Y., Li C., Hou Y., Feng M., Wang Y., Chen J. (2020). Integration of proteomics, lipidomics, and metabolomics reveals novel metabolic mechanisms underlying N, N-dimethylformamide induced hepatotoxicity. Ecotoxicol. Environ. Saf..

[135] Yuan P., Dong M., Lei H., Xu G., Chen G., Song Y., Ma J., Cheng L., Zhang L. (2020). Targeted metabolomics reveals that 2,3,7,8-tetrachlorodibenzofuran exposure induces hepatic steatosis in male mice. Environ. Pollut..

[136] O’Donnell V.B., Dennis E.A., Wakelam M.J.O., Subramaniam S. (2019). LIPID MAPS: Serving the next generation of lipid researchers with tools, resources, data, and training. Sci. Signal..

[137] Walker A.K., Jacobs R.L., Watts J.L., Rottiers V., Jiang K., Finnegan D.M., Shioda T., Hansen M., Yang F., Niebergall L.J. (2011). A Conserved SREBP-1/Phosphatidylcholine Feedback Circuit Regulates Lipogenesis in Metazoans. Cell.

[138] He J., Zhang F., Tay L.W.R., Boroda S., Nian W., Levental K.R., Levental I., Harris T.E., Chang J.T., Du G. (2017). Lipin-1 regulation of phospholipid synthesis maintains endoplasmic reticulum homeostasis and is critical for triple-negative breast cancer cell survival. FASEB J..

[139] Golden A., Liu J., Cohen-Fix O. (2009). Inactivation of the *C. elegans* lipin homolog leads to ER disorganization and to defects in the breakdown and reassembly of the nuclear envelope. J. Cell Sci..

[140] Jung Y., Kwon S., Ham S., Lee D., Park H.H., Yamaoka Y., Jeong D., Artan M., Altintas O., Park S. (2020). Caenorhabditis elegans Lipin 1 moderates the lifespan-shortening effects of dietary glucose by maintaining ω-6 polyunsaturated fatty acids. Aging Cell.

[141] Sapir A., Tsur A., Koorman T., Ching K., Mishra P., Bardenheier A., Podolsky L., Bening-Abu-Shach U., Boxem M., Chou T.-F. (2014). Controlled sumoylation of the mevalonate pathway enzyme HMGS-1 regulates metabolism during aging. Proc. Natl. Acad. Sci. USA.

[142] Chen J.-C., Wu M.-L., Huang K.-C., Lin W.-W. (2008). HMG-CoA reductase inhibitors activate the unfolded protein response and induce cytoprotective GRP78 expression. Cardiovasc. Res..

[143] Watts J.L., Ristow M. (2017). Lipid and Carbohydrate Metabolism in Caenorhabditis elegans. Genetics.

[144] Thinon E., Serwa R.A., Broncel M., Brannigan J.A., Brassat U., Wright M.H., Heal W.P., Wilkinson A.J., Mann D.J., Tate E.W. (2014). Global profiling of co- and post-translationally N-myristoylated proteomes in human cells. Nat. Commun..

[145] Thinon E., Morales-Sanfrutos J., Mann D.J., Tate E.W. (2016). N-Myristoyltransferase Inhibition Induces ER-Stress, Cell Cycle Arrest, and Apoptosis in Cancer Cells. ACS Chem. Biol..

[146] Schiavone M.L., Millucci L., Bernardini G., Giustarini D., Rossi R., Marzocchi B., Santucci A. (2020). Homogentisic acid affects human osteoblastic functionality by oxidative stress and alteration of the Wnt/β-catenin signaling pathway. J. Cell. Physiol..

[147] Fisher A.L., Page K.E., Lithgow G.J., Nash L. (2008). The Caenorhabditis elegans K10C2.4 Gene Encodes a Member of the Fumarylacetoacetate Hydrolase Family. J. Biol. Chem..

[148] Wang H., Chen H., Hao G., Yang B., Feng Y., Wang Y., Feng L., Zhao J., Song Y., Zhang H. (2013). Role of the Phenylalanine-Hydroxylating System in Aromatic Substance Degradation and Lipid Metabolism in the Oleaginous Fungus Mortierella alpina. Appl. Environ. Microbiol..

[149] Moseley K.D., Koch R., Moser A. (2002). Lipid Status and Long-Chain Polyunsaturated Fatty Acid Concentrations in Adults and Adolescents with Phenylketonuria on Phenylalanine-Restricted Diet. J. Inherit. Metab. Dis..

[150] Perkins R.J., Vaida V. (2017). Phenylalanine Increases Membrane Permeability. J. Am. Chem. Soc..

[151] Entchev E.V., Schwudke D., Zagoriy V., Matyash V., Bogdanova A., Habermann B., Zhu L., Shevchenko A., Kurzchalia T.V. (2008). LET-767 Is Required for the Production of Branched Chain and Long Chain Fatty Acids inCaenorhabditis elegans. J. Biol. Chem..

[152] Zhang H., Abraham N., Khan L.A., Hall D.H., Fleming J.T., Göbel V. (2011). Apicobasal domain identities of expanding tubular membranes depend on glycosphingolipid biosynthesis. Nat. Cell Biol..

[153] Kuervers L.M., Jones C.L., O’Neil N.J., Baillie D.L. (2003). The sterol modifying enzyme LET-767 is essential for growth, reproduction and development in Caenorhabditis elegans. Mol. Genet. Genom..

[154] Galles C., Prez G.M., Penkov S., Boland S., Porta E.O.J., Altabe S.G., Labadie G.R., Schmidt U., Knölker H.-J., Kurzchalia T.V. (2018). Endocannabinoids in Caenorhabditis elegans are essential for the mobilization of cholesterol from internal reserves. Sci. Rep..

[155] Garcia G. (2019). Lipid Homeostasis Is Essential for Endoplasmic Reticulum Protein Quality Control. Ph.D. Thesis.

[156] Kniazeva M., Crawford Q.T., Seiber M., Wang C.-Y., Han M. (2004). Monomethyl Branched-Chain Fatty Acids Play an Essential Role in Caenorhabditis elegans Development. PLoS Biol..

[157] Tam A.B., Roberts L.S., Chandra V., Rivera I.G., Nomura D., Forbes U.J., Niwa M. (2018). The UPR Activator ATF6 Responds to Proteotoxic and Lipotoxic Stress by Distinct Mechanisms. Dev. Cell.

[158] Micoogullari Y., Basu S.S., Ang J., Weisshaar N., Schmitt N.D., Abdelmoula W.M., Lopez B., Agar J.N., Agar N., Hanna J. (2020). Dysregulation of very-long-chain fatty acid metabolism causes membrane saturation and induction of the unfolded protein response. Mol. Biol. Cell.

[159] Williamson C.D., Wong D.S., Bozidis P., Zhang A., Colberg-Poley A.M. (2015). Isolation of Endoplasmic Reticulum, Mitochondria, and Mitochondria-Associated Membrane and Detergent Resistant Membrane Fractions from Transfected Cells and from Human Cytomegalovirus-Infected Primary Fibroblasts. Curr. Protoc. Cell Biol..

